# A Systematic Review Comparing the Outcomes of Cemented Versus Uncemented Stems in Femoral Impaction Bone Grafting for Revision Hip Arthroplasty

**DOI:** 10.7759/cureus.71560

**Published:** 2024-10-15

**Authors:** Khooi Zhong Yi, Veenesh Selvaratnam

**Affiliations:** 1 Graduate School of Medicine, Perdana University, Kuala Lumpur, MYS; 2 Joint Reconstruction Unit, National Orthopaedic Centre of Excellence for Research and Learning (NOCERAL) Department of Orthopaedic Surgery, Faculty of Medicine, Universiti Malaya, Kuala Lumpur, MYS

**Keywords:** aseptic loosening, cemented stems, complications, femoral impaction bone grafting, hip arthroplasty, systematic review, uncemented stems

## Abstract

Femoral impaction bone grafting is a crucial technique in revision hip arthroplasty, addressing bone loss and ensuring implant stability. The choice between cemented and uncemented stems significantly influences the outcomes and long-term success of the procedure. This systematic review aims to compare the clinical outcomes of cemented versus uncemented stems in femoral impaction bone grafting. A comprehensive search of PubMed, MEDLINE Complete, and the Cochrane Library databases was conducted in accordance with Preferred Reporting Items for Systematic Reviews and Meta-Analyses (PRISMA) guidelines. Studies evaluating the outcomes of femoral impaction bone grafting with cemented or uncemented stems were included. The primary outcome measured was the rate of loosening of the femoral component, while secondary outcomes included the incidence of complications such as dislocation, infection, fractures, overall patient mortality, and cardiopulmonary diseases. The literature search yielded 78 articles, with 36 meeting the inclusion criteria. These included one randomized controlled trial, 16 cohort studies (10 retrospective and six prospective), and 15 case series. Most surgeries were revision procedures, with aseptic loosening being the most common indication. For the cemented technique, 1,588 hips were analyzed, with 8.00% experiencing aseptic loosening, 3.53% dislocation, 3.87% infection, 37.33% mortality, 7.57% fractures, and 1.13% cardiopulmonary complications. For the uncemented technique, 464 hips were analyzed, with 1.72% aseptic loosening, 4.74% dislocation, 1.5% infection, 38.47% mortality, 7.76% fractures, and 0.65% cardiopulmonary complications. This systematic review highlights that both cemented and uncemented techniques for femoral impaction bone grafting offer unique benefits and challenges, with the choice depending on patient-specific factors. The uncemented technique, with a lower risk of femoral component loosening, may be better suited for younger, active patients with good bone quality, despite a slightly higher risk of fractures and dislocations. In contrast, the cemented technique, offering immediate stability, is more appropriate for elderly patients with compromised bone quality but carries a higher risk of loosening and cardiopulmonary complications. The decision should be tailored to the patient’s clinical profile, including age, bone quality, comorbidities, and the surgeon’s expertise.

## Introduction and background

**Figure 1:**
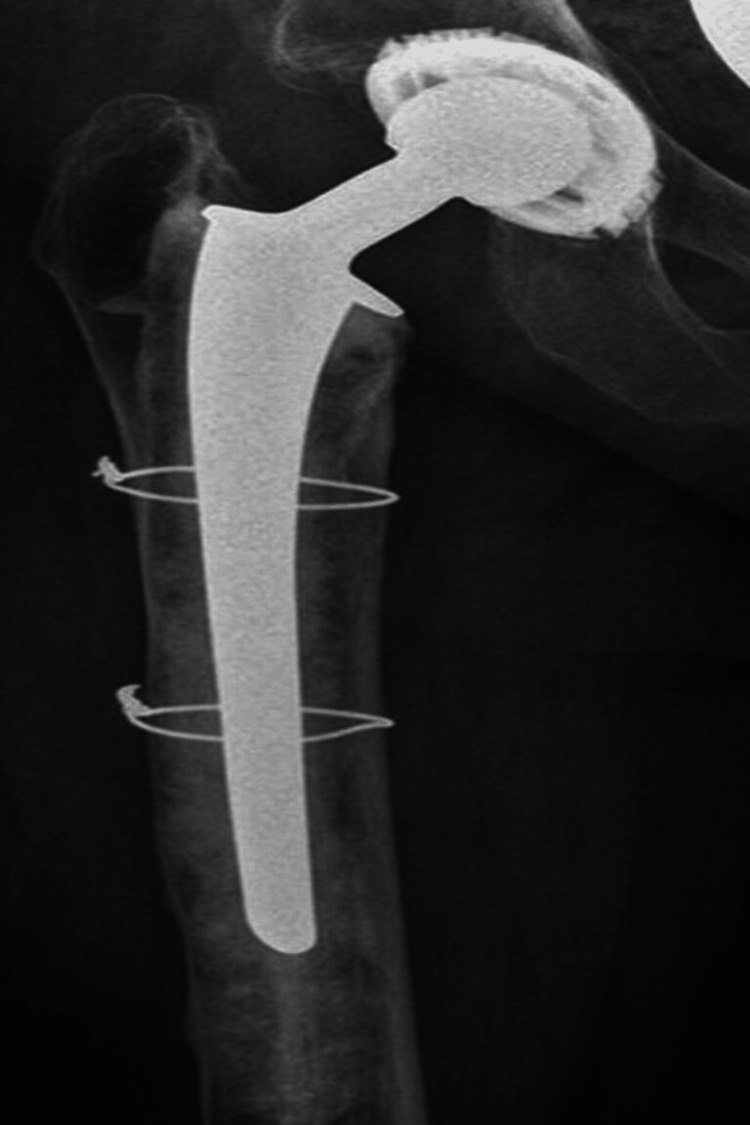
Cemented stem revision Follow-up radiograph two years after cemented stem revision with femoral impaction grafting. This image is reproduced with permission from Gehrke T, Gebauer M, Kendoff D. © 2013 British Editorial Society of Bone and Joint Surgery. All rights reserved [2].

Femoral impaction bone grafting (IBG) is a surgical technique employed in revision hip arthroplasty to address bone loss [1] and provide mechanical stability to the implant. The choice between cemented and uncemented stems is crucial in this procedure, as it significantly impacts its success. In cemented femoral grafting, a cemented prosthesis is commonly used. This involves applying a special bone cement, such as polymethylmethacrylate (PMMA), to fix the prosthesis to the femur. Figure 1 shows an illustration of a cemented prosthesis used in femoral IBG, showing the application of PMMA cement to fix the prosthesis to the femur, providing immediate stability and fixation.

**Figure 2:**
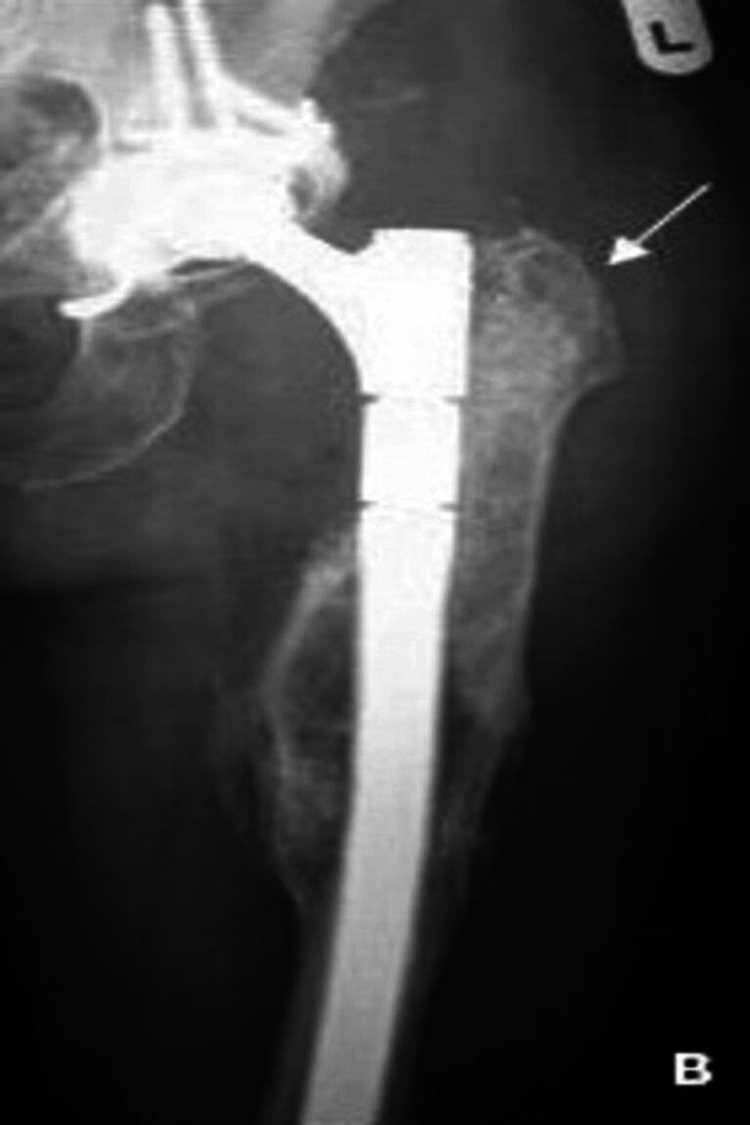
Uncemented stem Follow-up radiograph two years after a modular revision prosthesis titan uncemented stem revision with femoral impaction grafting and distal diaphyseal fixation. This image is reproduced from Wimmer MD, Randau TM, Deml MC, Ascherl R, Nöth U, Forst R, Gravius S. 2013; 14 [3].

On the other hand, Figure 2 shows an illustration of a non-cemented or press-fit prosthesis used in femoral IBG, illustrating the porous surface that allows for bone ingrowth and integration, providing long-term stability and fixation. These prostheses rely on the bone's natural healing process to gradually grow into the porous surface, providing long-term stability and fixation.

Advancements in modern fixation techniques, particularly those utilizing cement, have notably elevated outcomes compared to older cemented and historical uncemented series [4]. However, in recent years, developments in uncemented devices have showcased remarkable progress [5-8], demonstrating enhanced stability that now matches or even surpasses the reliability achieved through cemented fixation methods. This evolution underscores the dynamic nature of orthopedic practices: while traditional cemented approaches historically excelled, contemporary uncemented methods have emerged as robust alternatives, offering comparable levels of stability and promising long-term outcomes.

The initial method outlined by Gie et al. [9], with a relatively brief follow-up period, involved the utilization of cemented stems. Although some attempts have been made to employ uncemented stems alongside impaction grafting, the outcomes have generally been less positive [10]. Despite the generally less positive outcomes observed in this study, research by Masterson et al. [11] indicates no significant differences in outcomes or revision rates at an average follow-up of 9.8 years. This study comparing cemented (31 hips in 30 patients) and uncemented (33 hips in 30 patients) Freeman femoral components found no substantial disparities in outcomes or revision rates.

Existing research highlights the critical role of stem choice in femoral IBG outcomes, showcasing varied preferences, and emphasizing advantages and limitations in stability, longevity, and patient outcomes. However, gaps persist, including the insights gained within the initial two-year postoperative period, and there is an ongoing need for further investigation. This includes maintaining vigilance in understanding hip arthroplasty outcomes in the medium to long term after the two-year postoperative period, a vital avenue for future research.

## Review

Methodology

Search Strategy

This systematic review was conducted based on the Preferred Reporting Items for Systematic Reviews and Meta-Analyses (PRISMA) guidelines. The search string used in the literature search conducted in June 2023 was (femoral IBG) AND (cemented stems OR uncemented stems). Electronic databases, i.e., PubMed, MEDLINE Complete, and Cochrane Library were used in the literature search to identify relevant studies from 1987 to June 2023. All records from the inception of the databases were searched. Reference tracing of the included articles was conducted to confirm all relevant literature was included.

Inclusion/Exclusion Criteria

The inclusion criteria for this systematic review were studies that reported the outcomes of femoral IBG using cemented stems or uncemented stems. Studies published in the English language were included. On the other hand, studies that did not report the outcomes of interest were not in English, did not contain original data (e.g., letters, commentaries, or opinions), or animal studies were excluded from the review. Conference abstracts and proceedings were excluded to avoid duplication with the original articles.

Data Screening

The organization of literature and identification of duplicated items were performed using Endnote. Two independent reviewers screened the titles and abstracts of the identified studies to assess their relevance to the research questions. Full-text articles were obtained for the potentially relevant studies, and their eligibility was further assessed based on the inclusion and exclusion criteria. Any reviewer disagreements were resolved through discussion or consultation with a third reviewer.

Data Extraction

The extracted information included the following parameters: authors, mean age, indication for surgery, mean follow-up years, number of patients, and number of hips. Primary outcomes and secondary outcomes are presented in a table below. These data were categorized according to cemented and uncemented groups. This data was analyzed to identify trends, assess study quality, and evaluate the evidence on cemented and uncemented femoral IBG techniques. Limitations were considered to understand their impact on study validity. Data from the included studies will be extracted and analyzed using a random-effects model. The primary outcome will be the rate of loosening of the femoral component. Secondary outcomes will include dislocation, infection, mortality, fracture, and cardiopulmonary diseases.

Results

Search Results

**Figure 3:**
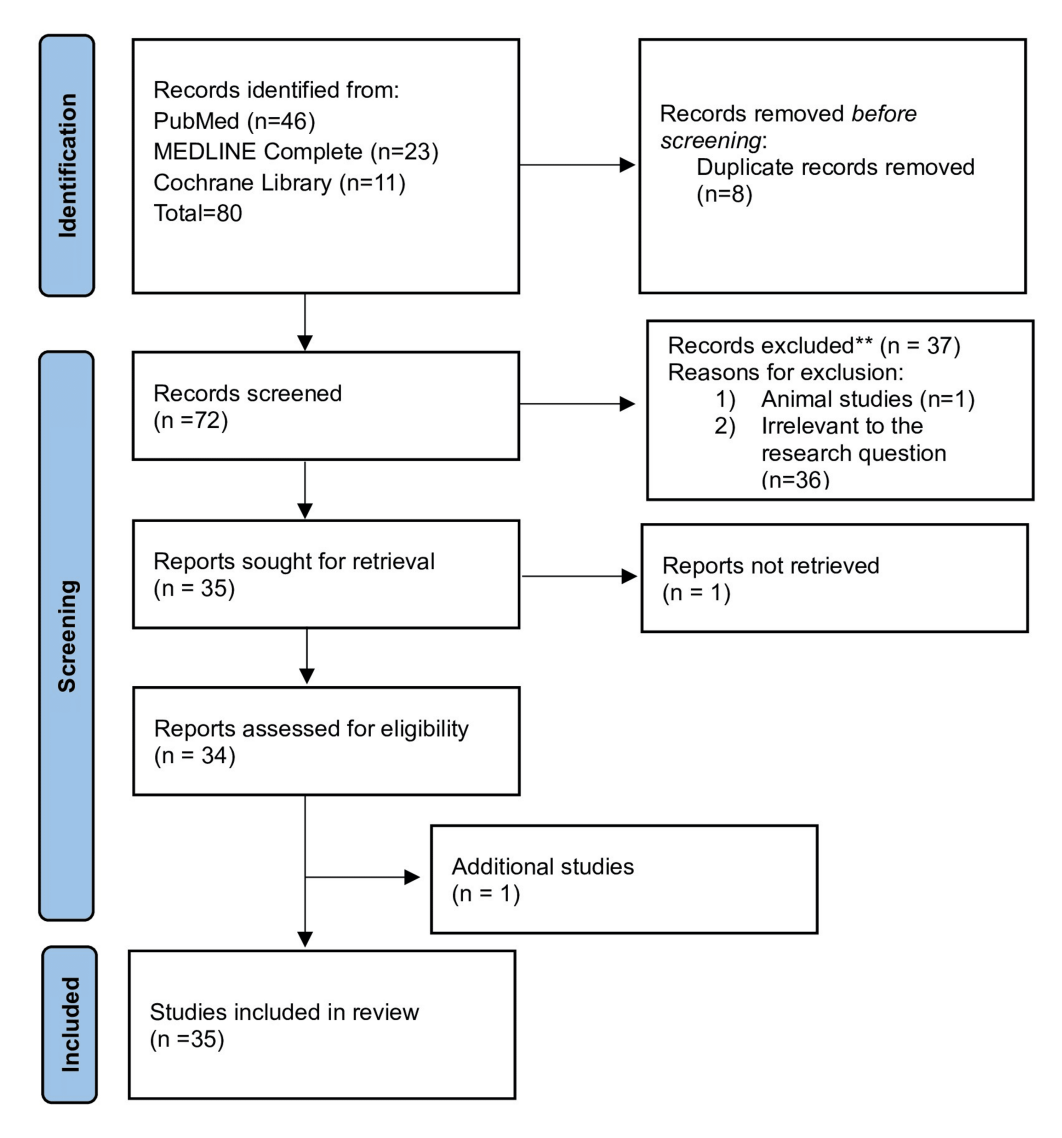
PRISMA flow diagram illustrating the search strategy and study selection process for the systematic review. PRISMA: Preferred Reporting Items for Systematic Reviews and Meta-Analyses Source: Page MJ, et al. BMJ 2021;372:n71. doi: 10.1136/bmj.n71.

The literature search revealed 78 articles (PubMed = 46, MEDLINE Complete = 23, and Cochrane Library = 11), of which seven were identified as duplicates and removed, and one was removed for other reasons. Of the remaining 70, only 35 of these met our criteria and were selected. However, the complete content of one of them has been removed from the affiliated journal. An additional publication was found through a manual search, giving a final total of 35 publications. The article screening and selection process is depicted in Figure 3.

Study Characteristics

The characteristics of the included studies are summarized in Table 1. Out of a total of 35 studies, one was a randomized controlled trial, 10 were retrospective cohort studies, five were prospective cohort studies, 15 were case series, and there was one of each for a review article, a case report, a longitudinal study, and a technical report. The sample size of patients ranged from 2 to 12,491. The mean age ranged from 46 to 83, while the follow-up period ranged from 7 months to 15 years. The quantities used in these results primarily refer to the number of hips; however, in some studies [12,13] only the number of patients was mentioned. Therefore, the results here used the number of patients in these two studies instead.

**Table 1:** Basic information from selected studies RCT: random controlled trial; RCS: retrospective cohort study; PCS: prospective cohort study; CS: case series; RA: review article; CR: case report; LS: longitudinal study; TR: technical report; NM: not mentioned; N/A: not applicable; UCH: uncemented hip replacement; CH: cemented hip replacement

Studies (First Author)	Type of Study	Mean Age (CH/UCH) (Years)	Indication for Surgery	Cemented Group				Uncemented Group			
											No. of patients	No. of hips	Mean-follow-up (years)	Cemented femur	No. of patients	No. of hips	Mean-follow-up period (years)		
				Rutherford et al.[12]	RCT	70 (UCH)	Degenerative native hip joint pathology (Index Surgery)	N/A	N/A	N/A	N/A	98	100	2 Years		
Boldt et al.[13]	RCS	71 (CH)	Early cup loosening	238	252	4	Yes	N/A	N/A	N/A		
Buttaro et al. [14]	CS	65 (CH)	Femoral defects	9	9	3.6	Yes	N/A	N/A	N/A		
											Chimutengwende-Gordon et al. [15]	TR	N/A										
Diaz-Dilernia et al.[16]	RCS	78(CH)/81(UCH)	Mainly OA (Index Surgery)	33	33	6.25	Yes	21	21	4.6													
											Elting et al. [17]	CS	65 (CH)	Femoral stem loosening	56	56	2.6	Yes	N/A	N/A	N/A		
Flugsrud et al. [18]	CS	74 (CH)	Mainly aseptic loosening	10	10	4	Yes	N/A	N/A	N/A		
Francés et al. [19]	RCS	68.5 (CH)	Aseptic femoral stem loosening	54	54	5.04	Yes	N/A	N/A	N/A		
Niikura [20]	CR	75.5 (CH)	Aseptic Loosening	2	2	2.04	Yes	N/A	N/A	N/A		
Garvin et al. [21]	RCS	67 (CH)	Mechanical loosening of the femoral component	71	78	10.5	Yes	N/A	N/A	N/A		
Halliday et al. [22]	RCS	NM	Mainly aseptic loosening	207	226	≥5	Yes	N/A	N/A	N/A		
Holt et al. [23]	RA	A Review Article										
Howie et al. [24]	PCS	63 (CH)	Mainly aseptic loosening	53	56	7.5	Yes	N/A	N/A	N/A		
te Stroet et al. [25]	PCS	66 (CH)	Mainly aseptic loosening	90	92	7.7	Yes	N/A	N/A	N/A		
Iwase et al. [26]	RCS	66.3 (CH)	Mainly aseptic loosening	97	103	5.2	Yes	N/A	N/A	N/A		
Kärrholm et al. [27]	CS	65 (CH)	Type I - III loosening	24	24	3	Yes	N/A	N/A	N/A		
Meding et al. [28]	CS	66.3 (CH)	Aseptic loosening	34	34	2.5	Yes	N/A	N/A	N/A		
Mikhail et al. [29]	CS	59 (CH)	Aseptic mechanical failure	40	43	6	Yes	N/A	N/A	N/A		
Nelissen et al. [30]	PCS	63 (CH)	Mainly RA	18	NM	2	Yes	N/A	N/A	N/A		
Nickelsen et al. [10]	CS	NM	Aseptic loosening	N/A	N/A	N/A	No	100	100	9.3		
Özdemir et al. [31]	PCS	64.9 (CH)	Mainly aseptic loosening	202	208	13.4	Yes	N/A	N/A	N/A		
Padgett and Kinkel [32]	CS	NM	Mainly failed cemented and uncemented THA	30	30	4	Yes	N/A	N/A	N/A		
Schreurs et al. [33]	PCS	63 (CH)	Mainly aseptic loosening	33	33	10.4	Yes	N/A	N/A	N/A		
Sierra et al. [34]	CS	73.8	Mainly aseptic loosening	40	42	7.5	Yes	N/A	N/A	N/A		
Sörensen et al. [35]	CS	68.8 (CH)	Loosening and osteolysis	5	5	1	Yes	N/A	N/A	N/A		
te Stroet et al. [36]	CS	76	Mainly aseptic loosening	37	37	9	Yes	N/A	N/A	N/A		
te Stroet et al. [37]	RCS	46.4 (CH)	Mainly aseptic loosening	33	34	11.7	Yes	N/A	N/A	N/A		
Tsiridis et al. [38]	RCS	68 (CH)	Periprosthetic femoral fractures	89	89	1	Yes	N/A	N/A	N/A		
Ullmark et al. [39]	CS	65.5 (CH)	Prosthetic loosening	5	5	6	Yes	N/A	N/A	N/A		
Van Kleunen et al. [40]	CS	63 (CH)	Failed hip arthroplasty (femoral defects)	17	18	0.7	Yes	N/A	N/A	N/A		
Verspeek et al. [41]	LS	46 (CH)	Mainly aseptic loosening	NM	33	15	Yes	N/A	N/A	N/A		
Wimmer et al. [3]	RCS	68 (UCH)	Bony defects of the proximal femur	N/A	N/A	N/A	N/A	233	243	4.4		
Yan et al. [42]	CS	61 (CH)	Mainly aseptic loosening	13	15	7.7	Yes	N/A	N/A	N/A		
Yim et al. [43]	CS	63 (CH)	Mainly aseptic loosening	43	56	4.03	N/A	N/A	N/A	N/A		
Okike et al. [44]	RCS	83 (CH and UCH)	Hip Fracture (Index Surgery)	6449	NM	3.8	Yes	6042	NM	3.8		

Type of Surgery and Preoperative Details

In the data collected from the studies reviewed, most surgeries were performed as revision procedures. However, only two studies [12,44] reported on index surgeries. Of the 36 studies of femoral IBG, two contain data exclusively on the uncemented method [10,12], two contain comparative data on both cemented and uncemented methods [16,44], and the remaining studies contain data exclusively on the cemented method. The data includes the predominant indication for surgery in each study. The most reported indication for revision femoral IBG using both cemented and uncemented methods was aseptic loosening. In the three studies of index surgeries, the primary diagnosis of femoral IBG includes degenerative native hip joint pathology [12] and hip fracture [44].

Outcomes

The outcome results are summarized in Table 2, detailing the primary outcome of femoral component loosening. Secondary outcomes, including dislocation, infection, mortality, fracture, and cardiopulmonary disease, are also presented for both cemented and uncemented groups.

**Table 2:** The rates and types of outcomes in cemented and uncemented groups NM: not mentioned; N/A: not applicable

Studies (First Author)	Mean Follow-up Period	Cemented						Uncemented						
		Loose femoral component	Dislocation	Infection	Mortality	Fracture	Cardiopulmonary Diseases	Loose femoral component	Dislocation	Infection	Mortality	Fracture	Cardiopulmonary Diseases	
Rutherford [12]	2 Years	N/A	N/A	N/A	N/A	N/A	N/A	1 (1%)	0	0	0	1 (%)	0	
Boldt et al. [13]	4 Years	4 (5.1%)	5 (2%)	3 (1.2%)	0	1 (0.4%)	NM	N/A	N/A	N/A	N/A	N/A	N/A	
Buttaro et al. [14]	3.6 Years	5 (55.5%)	2 (22.2%)	1 (11.1%)	0	9 (100%)	NM	N/A	N/A	N/A	N/A	N/A	N/A	
		Components were not specified.												
Chimutengwende-Gordon et al. [15]	NM													
														Diaz-Dilernia et al. [16]	6.25 Years (CH) / 4.6 Years (UCH)	5 (15%)	2 (6%)	4 (12%)	0	0	6 (18%), 4 DVT + 2 Pneumonia	1 (4.76%)	4 (19%)	0	0	0	1 (4.76%)		
Elting et al. [17]	2.6 Years	0	2 (6%)	4 (7.1%)	0	3 (5.4%)	4 (7.1%), pulmonary disease	N/A	N/A	N/A	N/A	N/A	N/A																
Flugsrud et al. [18]	4 Years	10 (100%)	1 (10%)	1 (10%)	0	1 (10%)	1 (10%)	N/A	N/A	N/A	N/A	N/A	N/A		
Francés et al. [19]	5.04 Years	0	8 (14.8%)	2 (3.7%)	0	2 (3.7%)	0	N/A	N/A	N/A	N/A	N/A	N/A		
Niikura [20]	2.04 Years	2 (100%)	0	0	0	1 (50%)	0	N/A	N/A	N/A	N/A	N/A	N/A		
Garvin et al. [21]	10.5 Years	1 (1.3%)	2 (2.6%)	2 (2.6%)	34 (47.9%)	2 (2.6%)	0	N/A	N/A	N/A	N/A	N/A	N/A		
Halliday et al. [22]	≥5 Years	16 (7.1%)	1 (0.4%)	5 (2.2%)	0	26 (11.5%)	0	N/A	N/A	N/A	N/A	N/A	N/A		
Holt et al. [23]	N/A														
Howie et al. [24]	7.5 Years	49 (87.5%)	0	4 (7.1%)	0	2 (3.6%)	0	N/A	N/A	N/A	N/A	N/A	N/A		
te Stroet et al. [25]	7.7 Years	0	12 (13%)	13 (14%)	2 (2.2%)	4 (4.3%)	1 (1.1%)	N/A	N/A	N/A	N/A	N/A	N/A		
Iwase et al. [26]	5.2 Years	1 (1%)	7 (6.8%)	2 (1.9%)	0	25 (24.3%)	2 (1.9%)	N/A	N/A	N/A	N/A	N/A	N/A		
Kärrholm et al. [27]	3 Years	0	0	0	0	0	0	N/A	N/A	N/A	N/A	N/A	N/A		
Meding et al. [28]	2.5 Years	2 (5.9%)	1 (2.9%)	0	0	6 (17.7%)	0	N/A	N/A	N/A	N/A	N/A	N/A		
Mikhail et al. [29]	6 Years	0	3 (7%)	1 (2.3%)	0	2 (4.7%)	0	N/A	N/A	N/A	N/A	N/A	N/A		
Nelissen et al. [30]	2 Years	NM	NM	NM	1 (5.6%)	NM	NM	N/A	N/A	N/A	N/A	N/A	N/A		
Nickelsen et al. [10]	9.3 Years	N/A	N/A	N/A	N/A	N/A	N/A	0	3 (3%)	3 (3%)	33 (33%)	14 (14%)	2 (2%)		
Özdemir et al. [31]	13.4 Years	0	0	0	0	4 (12.1%)	0	N/A	N/A	N/A	N/A	N/A	N/A		
Padgett and Kinkel [32]	4 Years	1 (3.3%)	0	1 (3.3%)	0	0	0	N/A	N/A	N/A	N/A	N/A	N/A		
Schreurs et al. [33]	10.4 Years	2 (6.1%)	0	0	1 (3%)	4 (12.1%)	0	N/A	N/A	N/A	N/A	N/A	N/A		
Sierra et al. [34]	7.5 Years	1 (2.4%)	4 (9.5%)	5 (11.9%)	1 (2.4%)	2 (4.8%)	1 (2.4%)	N/A	N/A	N/A	N/A	N/A	N/A		
Sörensen et al. [35]	1 Year	NM						N/A	N/A	N/A	N/A	N/A	N/A		
te Stroet et al. [36]	9 Years	0	3 (8.1%)	4 (10.8%)	14 (37.8%)	2 (4.8%)	0	N/A	N/A	N/A	N/A	N/A	N/A		
te Stroet et al. [37]	11.7 Years	4 (11.8%)	2 (5.9%)	4 (11.8%)	0	1 (2.9%)	0	N/A	N/A	N/A	N/A	N/A	N/A		
Tsiridis et al. [38]	1 Year	NM	NM	4 (4.5%)	NM	15 (16.9%)	NM	N/A	N/A	N/A	N/A	N/A	N/A		
Ullmark et al. [39]	6 Years	NM						N/A	N/A	N/A	N/A	N/A	N/A		
Van Kleunen et al. [40]	0.7 Year	4 (22.2%)	0	0	0	1 (5.6%)	0	N/A	N/A	N/A	N/A	N/A	N/A		
Verspeek et al. [41]	15 Years	10 (30.3%)	1 (3%)	3 (9.1%)	0	0	0	N/A	N/A	N/A	N/A	N/A	N/A		
Wimmer et al. [3]	4.4 Years	N/A	N/A	N/A	N/A	N/A	N/A	6 (2.5%)	15 (6.2%)	4 (1.6%)	0	21 (8.6%)	0		
Yan et al. [42]	7.7 Years	0	0	2 (13.3%)	0	6 (40%)	0	N/A	N/A	N/A	N/A	N/A	N/A		
Yim et al. [43]	4.03 Years	0	0	0	0	5 (9%)	0	N/A	N/A	N/A	N/A	N/A	N/A		
Okike et al. [44]	4.03 Years	NM	NM	NM	2953 (45.8%)	NM	NM	NM	NM	NM	2470 (40.9%)	NM	NM		

In the analysis of the cemented technique, a total of 1,578 hips/patients were evaluated after deducting the number of hips in studies that did not mention the loosening of femoral components [30,35,38,39,44]. Among these, 117 hips (7.4%) experienced aseptic loosening. In the uncemented technique, a total of 464 hips/patients were evaluated after deducting the number of hips in studies that did not mention the loosening of femoral components [44]. Among these, eight hips (1.72%) experienced aseptic loosening.

In the cemented technique, a total of 1,578 hips/patients were evaluated after excluding the studies that did not mention dislocation [30,35,38,39,44]. Among these, 56 hips/patients (3.55%) experienced dislocation. In the uncemented technique, a total of 464 hips/patients were evaluated after excluding the study [44]. Among these, 22 hips/patients (4.74%) experienced dislocation.

In the cemented technique, a total of 1,667 hips/patients were evaluated after excluding the studies that did not mention infection [30,35,39,44]. Among these, 65 hips/patients (3.9%) experienced infection. In the uncemented technique, a total of 464 hips/patients were evaluated after excluding the study [44]. Among these, seven hips/patients (1.5%) experienced infection.

For the cemented technique, a total of 8,045 hips/patients were evaluated after excluding the studies [35,38,39] that did not mention the mortality rate. Among these, 3,006 hips/patients (37.36%) resulted in mortality. For the uncemented technique, a total of 6,506 hips/patients were evaluated with no studies excluded. Among these, 2,503 hips/patients (38.47%) resulted in mortality.

Both the cemented and uncemented techniques include intraoperative and postoperative fractures. For the cemented technique, a total of 1,667 hips/patients were evaluated after excluding the studies [30,35,39,44] that did not mention fractures. Among these, 124 hips/patients (7.44%) experienced fractures. For the uncemented technique, a total of 464 hips/patients were evaluated after excluding the study [44]. Among these, 36 hips/patients (7.76%) experienced fractures.

For the cemented technique, a total of 1,317 hips/patients were evaluated after excluding the studies [13,14,30,35,39,44] that did not mention cardiopulmonary disease. Among these, 15 hips/patients (1.14%) experienced cardiopulmonary disease. For the uncemented technique, a total of 464 hips/patients were evaluated after excluding the study [44]. Among these, three hips/patients (0.65%) experienced cardiopulmonary disease.

Discussion

This systematic review evaluated the comparative outcomes of cemented and uncemented stems in femoral IBG, focusing on primary and secondary outcomes such as implant failure, dislocation, infection, mortality, fracture, and cardiopulmonary disease. Both techniques encompass intraoperative and postoperative considerations, making it essential to assess their long-term efficacy and safety.

The results indicate that while the cemented technique is effective, a significant incidence of femoral component loosening could impact the procedure's overall success. A Swedish study [45] by Hierton et al. highlights that surgical outcomes are heavily influenced by the surgeon's experience and expertise. Experienced surgeons tend to achieve better results due to their knowledge and skill. In contrast, less experienced surgeons are more likely to encounter femoral component loosening, especially when the cement does not surround the components. Specifically, incomplete cement coverage of the femoral components, either proximally or distally, is significantly (P < 0.001) more common in hips operated on by less experienced surgeons. Despite the expertise of experienced surgeons, loosening still occurs, indicating that factors such as cement type and prosthesis design also play a critical role. In cases involving less experienced surgeons, the impact of poor cementation is even more pronounced.

When comparing the two techniques, the uncemented method demonstrates a significantly lower rate of femoral component loosening. This is likely due to differences in biomechanical stability, as cementless hip fixation methods can withstand biomechanical loading forces greater than full-weight bearing [46].

The cemented technique, which uses PMMA to fix the prosthesis, showed a slightly lower fracture rate (7.44%) compared to the uncemented technique (7.76%). This suggests that while the immediate stability provided by cement might be beneficial, it does not necessarily result in a lower long-term fracture risk. The uncemented technique, relying on bone ingrowth and integration, had a slightly higher fracture rate, which could indicate that patients with uncemented arthroplasty may be more susceptible to trauma during the first postoperative year. It is highly probable that some fractures associated with the uncemented method occur during the index operation but go unnoticed at the time of surgery. This hypothesis is supported by a study [47] by Fernandez, et al. showing a high incidence of intraoperative fractures. The study suggested that the intraoperative femoral fractures in cementless hip arthroplasty may result from the surgeon's excessive efforts to achieve an optimal fit and fill, or from issues with the design of the instruments or implants.

In terms of cardiopulmonary complications, the cemented technique had a higher incidence (1.14%) compared to the uncemented technique (0.65%). This finding aligns with existing literature [48] suggesting that cemented techniques, specifically the implantation of acrylic bone cement into the femur, can increase plasma histamine levels by more than 1 ng/ml. Although this may seem minimal, it can lead to serious, sometimes fatal cardiovascular complications in patients with pre-existing cardiac conditions or hypovolemia. Some studies have indicated an elevated risk of acute cardiopulmonary complications, venous thromboembolism, and mortality associated with the use of cement during total hip arthroplasty [49,50]. However, this complication is exceedingly rare and can potentially be minimized through modifications in technique to reduce intramedullary pressure [51], which aligns with the results of our review showing that cardiopulmonary complications have the least occurrence among all secondary outcomes.

The mortality rates for both techniques were notably high, with the cemented technique at 37.36% and the uncemented technique at 38.47%. These figures reflect the high-risk population typically undergoing revision hip arthroplasty, often with multiple comorbidities. A study [44] by Okike et al. included in the review, which contributed to the highest number of mortalities for both techniques, highlighted medical comorbidities such as chronic kidney disease, diabetes, and chronic pulmonary disease. The similarity in mortality rates suggests that the choice of cemented versus uncemented stems may not significantly influence long-term survival outcomes.

Regarding dislocation and infection rates, the cemented technique had lower dislocation (3.55%) and higher infection (3.9%) rates compared to the uncemented technique (4.74% and 1.5%, respectively). This suggests that while cemented stems might provide more immediate stability [52], they may carry a higher infection risk, possibly due to the presence of bone cement, which can harbor bacteria. However, a study [53] from the Swedish Hip Arthroplasty Register found that uncemented stems had a significantly higher risk of dislocation during the first three years post-surgery compared to cemented stems (HR 5, CI 1.2-23). This early instability in uncemented stems could be related to the reliance on bone ingrowth for fixation, which takes time to achieve optimal stability. Despite this early risk, the long-term outcomes for dislocation between the two techniques appear to converge, as the risk of re-revision due to dislocation becomes similar after three years (HR 0.5, CI 0.2-1.4). One study indicates that uncemented hip arthroplasties have a similar risk of revision due to infection as cemented arthroplasties with antibiotic-loaded cement, but a lower risk compared to cemented arthroplasties without antibiotic-loaded cement [54]. Necrotic bone tissue around the cement, due to either the cement's toxicity or the heat produced while it sets, could become a potential growth environment [55-57]. Antibiotic-loaded cement may provide prophylactic benefits in reducing infection risk during femoral impaction grafting. However, the dosage must be carefully controlled to prevent mechanical complications from the antibiotics and to avoid potential renal failure [58]. Additionally, dislocation rates in both techniques are influenced by multifactorial factors, such as the size of the femoral head. For instance, the use of 32-mm femoral heads has been shown to reduce revisions for dislocation substantially compared to 22- to 28-mm heads [59]. Furthermore, studies [52,59] by Stralen, et al. and Zijlstra, et al. suggest that the posterolateral approach in THA has a lower risk of dislocation compared to the direct anterior and anterolateral approaches. Therefore, combining the use of a larger femoral head, such as 32 mm or better yet 36 mm, with the posterolateral approach could significantly reduce the risk of dislocation.

The choice between cemented and uncemented stems in femoral IBG should be individualized based on patient-specific factors, including bone quality, comorbidities, and the surgeon’s expertise. While the higher fracture and dislocation rates in uncemented techniques are important considerations, a recent meta-analysis [60] found no significant differences in dislocation rates between cemented and uncemented long stems after mid-term follow-up (RR = 0.50; 95% CI: 0.10-2.47; P = 0.39). This suggests that the observed differences may depend on other factors, such as surgical technique or early postoperative care. However, the lower cardiopulmonary complication rates associated with uncemented techniques should also be considered, as the use of acrylic bone cement in cemented procedures can pose potential risks. Ultimately, the decision should be tailored to the individual patient's circumstances, balancing the risks and benefits of each approach.

This review has several limitations. The heterogeneity in study design and variation in follow-up periods could introduce variability in the reported outcomes and affect the comparability of long-term results. Differences in sample sizes and potential biases related to surgeon experience may also impact the generalizability of the findings. Additionally, the number of studies on the cemented technique far exceeds those on the uncemented one, leading to a potential imbalance in data interpretation. Furthermore, the review did not account for the type of surgical approach, cement, or bone grafting technique used, which could influence the outcomes and limit the comprehensiveness of the analysis. A further limitation is that not all included studies reported p-values or confidence intervals for the assessed outcomes, which restricts the ability to conduct precise statistical comparisons.

## Conclusions

This systematic review demonstrates that both cemented and uncemented techniques for femoral IBG offer distinct benefits and challenges, with specific considerations for different patient populations. The uncemented technique, which shows a lower incidence of femoral component loosening, may be more suitable for younger, more active patients with good bone quality. However, this technique is associated with a slightly higher risk of fractures and dislocations, necessitating close postoperative monitoring.

Conversely, the cemented technique, providing immediate stability, may be better suited for elderly patients with compromised bone quality, although it carries a higher risk of femoral component loosening and cardiopulmonary complications. Given the increased risk of cardiopulmonary issues with the cemented method, the uncemented technique is preferable for patients with pre-existing cardiopulmonary conditions. Additionally, while the cemented technique has a slightly higher rate of infections, its immediate stability can be advantageous in preventing other complications. Ultimately, the choice between cemented and uncemented techniques should be tailored to the individual patient’s clinical profile, considering factors such as age, bone quality, risk of loosening or infection, comorbidities, and the surgeon’s expertise.

## References

[1] Jones SA (2017). Impaction grafting made easy. J Arthroplasty.

[2] Gehrke T, Gebauer M, Kendoff D (2013). Femoral stem impaction grafting: Extending the role of cement. Bone Joint J.

[3] Wimmer MD, Randau TM, Deml MC (2013). Impaction grafting in the femur in cementless modular revision total hip arthroplasty: A descriptive outcome analysis of 243 cases with the MRP-TITAN revision implant. BMC Musculoskelet Disord.

[4] Thanner J (2022). The acetabular component in total hip arthroplasty: Evaluation of different fixation principles. Acta Orthopaedica Scandinavica. Supplementum.

[5] Zicat B, Engh CA, Gokcen E (1995). Patterns of osteolysis around total hip components inserted with and without cement. J Bone Joint Surg Am.

[6] Kim YH, Kim JS, Cho SH (1999). Primary total hip arthroplasty with the AML total hip prosthesis. Clin Orthop Relat Res.

[7] Della Valle CJ, Berger RA, Shott S, Rosenberg AG, Jacobs JJ, Quigley L, Galante JO (2004). Primary total hip arthroplasty with a porous-coated acetabular component: A concise follow-up of a previous report. J Bone Joint Surg Am.

[8] Sinha RK, Dungy DS, Yeon HB (2004). Primary total hip arthroplasty with a proximally porous-coated femoral stem. J Bone Joint Surg Am.

[9] Gie GA, Linder L, Ling S, Simon JP, Slooff TJ, Timperley AJ (1993). Impacted cancellous allografts and cement for revision total hip arthroplasty. J Bone Joint Surg.

[10] Nickelsen TN, Erenbjerg M, Retpen JB, Solgaard S (2008). Femoral revision with impaction allografting and an uncemented femoral component. Hip Int.

[11] Masterson S, Lidder S, Scott G (2012). Impaction femoral allografting at revision hip arthroplasty: Uncemented versus cemented technique using a Freeman femoral component. J Bone Joint Surg Br.

[12] Rutherford M, Khan RJ, Fick DP, Haebich S, Nivbrant O, Kozak T (2019). Randomised clinical trial assessing migration of uncemented primary total hip replacement stems, with and without autologous impaction bone grafting. Int Orthop.

[13] Boldt JG, Dilawari P, Agarwal S, Drabu KJ (2001). Revision total hip arthroplasty using impaction bone grafting with cemented nonpolished stems and Charnley cups. J Arthroplast.

[14] Buttaro M, Comba F, Zanotti G (2015). Fracture of the C-stem cemented femoral component in revision hip surgery using bone impaction grafting technique: Report of 9 cases. HIP International Website.

[15] Chimutengwende-Gordon M, Baker MP, Jagiello J (2021). Cemented revision hip arthroplasty with femoral impaction bone grafting. J Am Acad Orthop Surg.

[16] Diaz-Dilernia F, Slullitel PA, Oñativia JI, Comba FM, Piccaluga F, Buttaro MA (2019). Impaction bone grafting or uncemented modular stems for the treatment of type B3 periprosthetic femoral fractures? A complication rate analysis. J Arthroplasty.

[17] Elting JJ, Zicat BA, Mikhail WE, Hubbell JC, House BS (2013). Impaction grafting: Preliminary report of a new method for exchange femoral arthroplasty. Orthopedics.

[18] Flugsrud GB, Ovre S, Grøgaard B, Nordsletten L (2000). Cemented femoral impaction bone grafting for severe osteolysis in revision hip arthroplasty. Good results at 4-year follow-up of 10 patients. Arch Orthop Trauma Surg.

[19] Francés A, Moro E, Cebrian JL, Marco F, García-López A, Serfaty D, López-Durán L (2007). Reconstruction of bone defects with impacted allograft in femoral stem revision surgery. Int Orthop.

[20] Niikura T (2008). Histologic analysis of allograft mixed with hydroxyapatite-tricalcium phosphate used in revision femoral impaction bone grafting. Orthopedics.

[21] Garvin KL, Konigsberg BS, Ommen ND, Lyden ER (2013). What is the long-term survival of impaction allografting of the femur?. Clin Orthop Relat Res.

[22] Halliday BR, Timperley AJ, English HW, Gie GA, Ling RS (2003). Femoral impaction grafting with cement in revision total hip replacement: Evolution of the technique and results. J Bone Joint Surg.

[23] Holt G, Hook S, Hubble M (2011). Revision total hip arthroplasty: The femoral side using cemented implants. Int Orthop.

[24] Howie DW, Callary SA, McGee MA (2010). Reduced femoral component subsidence with improved impaction grafting at revision hip arthroplasty. Clin Orthop Relat Res.

[25] Te Stroet MA, Rijnen WH, Gardeniers JW, Van Kampen A, Schreurs BW (2015). Medium-term follow-up of 92 femoral component revisions using a third-generation cementing technique. Acta Orthop.

[26] Iwase T, Otsuka H, Katayama N, Fujita H (2012). Impaction bone grafting for femoral revision hip arthroplasty with Exeter Universal stem in Japan. Arch Orthop Trauma Surg.

[27] Kärrholm J, Hultmark P, Carlsson L, Malchau H (1999). Subsidence of a non-polished stem in revisions of the hip using impaction allograft. J Bone Joint Surg.

[28] Meding JB, Ritter MA, Keating EM, Faris PM, Mooresville I (1997). Impaction bone-grafting before insertion of a femoral stem with cement in revision total hip arthroplasty. A minimum two-year follow-up study. J Bone Joint Surg.

[29] Mikhail M, Wretenberg PF, Weidenhielm LR, Mikhail MN (1999). Complex cemented revision using polished stem and morselized allograft. Arch Orthop Trauma Surg.

[30] Nelissen RG, Valstar ER, Pöll RG, Garling EH, Brand R (2002). Factors associated with excessive migration in bone impaction hip revision surgery: A radiostereometric analysis study. J Arthroplasty.

[31] Özdemir E, Kuijpers MF, Visser J, Schreurs BW, Rijnen WH (2022). Thirty years of experience with instrumented femoral impaction bone grafting and a cemented polished Exeter stem: A prospective cohort study of 208 revision arthroplasties performed between 1991 and 2007. Bone Joint J.

[32] Padgett DE, Kinkel S (2011). Cancellous impaction grafting in femoral revision THA. Orthopedics.

[33] Schreurs BW, Arts JJ, Verdonschot N, Buma P, Slooff TJ, Gardeniers JW (2005). Femoral component revision with use of impaction bone-grafting and a cemented polished stem. J Bone Joint Surg Am.

[34] Sierra RJ, Charity J, Tsiridis E, Timperley JA, Gie GA (2008). The use of long cemented stems for femoral impaction grafting in revision total hip arthroplasty. J Bone Joint Surg Am.

[35] Sörensen J, Ullmark G, Långström B, Nilsson O (2003). Rapid bone and blood flow formation in impacted morselized allografts: Positron emission tomography (PET) studies on allografts in 5 femoral component revisions of total hip arthroplasty. Acta Orthop Scand.

[36] Te Stroet MA, Bronsema E, Rijnen WH, Gardeniers JW, Schreurs BW (2014). The use of a long stem cemented femoral component in revision total hip replacement: A follow-up study of five to 16 years. Bone Joint J.

[37] te Stroet MA, Rijnen WH, Gardeniers JW, van Kampen A, Schreurs BW (2015). Satisfying outcomes scores and survivorship achieved with impaction grafting for revision THA in young patients. Clin Orthop Relat Res.

[38] Tsiridis E, Narvani AA, Haddad FS, Timperley JA, Gie GA (2004). Impaction femoral allografting and cemented revision for periprosthetic femoral fractures. J Bone Joint Surg Br.

[39] Ullmark G, Sörensen J, Långström B, Nilsson O (2007). Bone regeneration 6 years after impaction bone grafting: A PET analysis. Acta Orthop.

[40] Van Kleunen JP, Anbari KK, Vu D, Garino JP (2006). Impaction allografting for massive femoral defects in revision hip arthroplasty using collared textured stems. J Arthroplasty.

[41] Verspeek J, Nijenhuis TA, Kuijpers MF, Rijnen WH, Schreurs BW (2021). what are the long-term results of cemented revision THA with use of both acetabular and femoral impaction bone grafting in patients younger than 55 years?. Clin Orthop Relat Res.

[42] Yan CH, Chiu KY, Ng TP, Ng FY (2010). Revision total hip arthroplasty with femoral impaction bone grafting. J Orthop Surg.

[43] Yim SJ, Kim MY, Suh YS (2007). Impaction allograft with cement for the revision of the femoral component. A minimum 39-month follow-up study with the use of the Exeter stem in Asian hips. Int Orthop.

[44] Okike K, Chan PH, Prentice HA, Paxton EW, Burri RA (2020). Association between uncemented vs cemented hemiarthroplasty and revision surgery among patients with hip fracture. JAMA.

[45] Hierton C, Blomgren G, Lindgren U (1983). Factors associated with early loosening of cemented total hip prostheses. Acta Orthop Scand.

[46] Kußmaul AC, Bruder J, Greiner A (2024). Uncemented hip revision cup as an alternative for T-type acetabular fractures: A cadaveric study. Orthop Traumatol Surg Res.

[47] Fernandez-Fernandez R, García-Elias E, Gil-Garay E (2007). Peroperative fractures in uncemented total hip arthrography: Results with a single design of stem implant. Int Orthop.

[48] Tryba M, Thole H, Wruck G (1987). Cardiovascular reactions due to histamine release during bone-cement implantation for total hip joint replacement. Anesthesiology.

[49] Sharrock NE, Cazan MG, Hargett MJ, Williams-Russo P, Wilson PD Jr (1995). Changes in mortality after total hip and knee arthroplasty over a ten-year period. Anesth Analg.

[50] Ries MD, Lynch F, Rauscher LA, Richman J, Mick C, Gomez M (1993). Pulmonary function during and after total hip replacement. Findings in patients who have insertion of a femoral component with and without cement. J Bone Joint Surg.

[51] Parvizi J, Holiday AD, Ereth MH, Lewallen DG (1999). The Frank Stinchfield Award. Sudden death during primary hip arthroplasty. Clin Orthop Relat Res.

[52] van Stralen GM, Struben PJ, van Loon CJ (2003). The incidence of dislocation after primary total hip arthroplasty using posterior approach with posterior soft-tissue repair. Arch Orthop Trauma Surg.

[53] Tyson Y, Hillman C, Majenburg N (2021). Uncemented or cemented stems in first-time revision total hip replacement? An observational study of 867 patients including assessment of femoral bone defect size. Acta Orthop.

[54] Engesæter LB, Espehaug B, Lie SA (2006). Does cement increase the risk of infection in primary total hip arthroplasty? Revision rates in 56,275 cemented and uncemented primary THAs followed for 0-16 years in the Norwegian Arthroplasty Register. Acta Orthopaedica.

[55] Davis N, Curry A, Gambhir AK (1999). Intraoperative bacterial contamination in operations for joint replacement. J Bone Joint Surg.

[56] Clarke MT, Lee PT, Roberts CP, Gray J, Keene GS, Rushton N (2004). Contamination of primary total hip replacements in standard and ultra-clean operating theaters detected by the polymerase chain reaction. Acta Orthop Scand.

[57] Maathuis PG, Neut D, Busscher HJ, van der Mei HC, van Horn JR (2005). Perioperative contamination in primary total hip arthroplasty. Clin Orthop Relat Res.

[58] Khanna G, Cernovsky J (2012). Bone cement and the implications for anaesthesia. Cont Educ Anaes Crit Care Pain.

[59] Zijlstra WP, De Hartog B, Van Steenbergen LN, Scheurs BW, Nelissen RG (2017). Effect of femoral head size and surgical approach on risk of revision for dislocation after total hip arthroplasty. Acta Orthop.

[60] Elbardesy H, Anazor F, Mirza M, Aly M, Maatough A (2023). Cemented versus uncemented stems for revision total hip replacement: A systematic review and meta-analysis. World J Orthop.

